# Inhibition of BCR signaling using the Syk inhibitor TAK-659 prevents stroma-mediated signaling in chronic lymphocytic leukemia cells

**DOI:** 10.18632/oncotarget.13557

**Published:** 2016-11-24

**Authors:** Noelia Purroy, Júlia Carabia, Pau Abrisqueta, Leire Egia, Meritxell Aguiló, Cecilia Carpio, Carles Palacio, Marta Crespo, Francesc Bosch

**Affiliations:** ^1^ Laboratory of Experimental Hematology, Department of Hematology, Vall d'Hebron University Hospital, Universitat Autònoma de Barcelona, Barcelona, Spain

**Keywords:** CLL, Syk, microenvironment, TAK-659, BCR inhibitor

## Abstract

Proliferation and survival of chronic lymphocytic leukemia (CLL) cells depend on microenvironmental signals coming from lymphoid organs. One of the key players involved in the crosstalk between CLL cells and the microenvironment is the B-cell receptor (BCR). Syk protein, a tyrosine kinase essential for BCR signaling, is therefore a rational candidate for targeted therapy in CLL. Against this background, we tested the efficacy of the highly specific Syk inhibitor TAK-659 in suppressing the favorable signaling derived from the microenvironment. To *ex vivo* mimic the microenvironment found in the proliferation centers, we co-cultured primary CLL cells with BM stromal cells (BMSC), CD40L and CpG ODN along with BCR stimulation. In this setting, TAK-659 inhibited the microenvironment-induced activation of Syk and downstream signaling molecules, without inhibiting the protein homologue ZAP-70 in T cells. Importantly, the pro-survival, proliferative, chemoresistant and activation effects promoted by the microenvironment were abrogated by TAK-659, which furthermore blocked CLL cell migration toward BMSC, CXCL12, and CXCL13. Combination of TAK-659 with other BCR inhibitors showed synergistic effect in inducing apoptosis, and the sequential addition of TAK-659 in ibrutinib-treated CLL cells induced significantly higher cytotoxicity. These findings provide a strong rationale for the clinical development of TAK-659 in CLL.

## INTRODUCTION

Chronic lymphocytic leukemia (CLL) is characterized by the expansion of monoclonal, mature CD5^+^/CD23^+^ B cells in the peripheral blood (PB) [1], secondary lymphoid tissues, and the bone marrow (BM).In this microenvironment CLL cells receive survival, proliferation and drug resistance signals from accessory cells and soluble factors [2]. The B-cell receptor (BCR) is one of the main molecules involved in this cross-talk, playing a critical role in CLL pathogenesis and prognosis [3]. The importance of the BCR signaling is underlined by several observations. First, from the clinical standpoint, time to first therapy, progression free and overall survival in CLL is dictated in part by whether *IGHV* genes have undergone somatic hypermutation (M-CLL) or not (U-CLL) [1]. Of note, U-CLL cells have stronger BCR activation and increased proliferation, linking BCR signaling to clinical progression [4]. Moreover, the clinical relevance of BCR signaling has also been inferred by the prognostic impact of ZAP-70 expression. This protein is associated with an increased BCR signaling in CLL cells [5], which translates into an enhanced ability to respond to survival and migratory signals [6]. Finally, the relevance of the BCR signaling in CLL has been proved by the demonstration of an extraordinary clinical activity of several inhibitors of key downstream kinases, such as ibrutinib, idelalisib, duvelisib and many others [7, 8].

Signal transduction initiated by BCR activation leads to the recruitment, phosphorylation, and sustained activity of the spleen tyrosine kinase (Syk) [9]. In CLL, Syk has been shown to be up-regulated at both the mRNA and protein levels, [10] and a constitutive Syk activation has been described [11]. Therefore, Syk has been hypothesized to be an excellent candidate for targeted therapy in CLL. The effect of Syk inhibition has been tested with fostamatinib (R406), a kinase inhibitor with limited specificity to Syk, demonstrating induction of apoptosis and blockade of chemokine-induced migration of CLL cells [11, 12] Fostamatinib has been clinically evaluated in CLL and other B cell malignancies with a hint of efficacy in these diseases [13, 14]. Herein, we presented the effectiveness of the novel, highly specific Syk inhibitor TAK-659 in suppressing the induction of survival, proliferation and migration of CLL cells by the microenvironment, thus providing the biological rationale for its clinical development in CLL.

## RESULTS

### BCR stimulation increases viability and enhances proliferation in primary CLL cells co-cultured with BMSC, CD40L and CpG ODN

**Figure 1:**
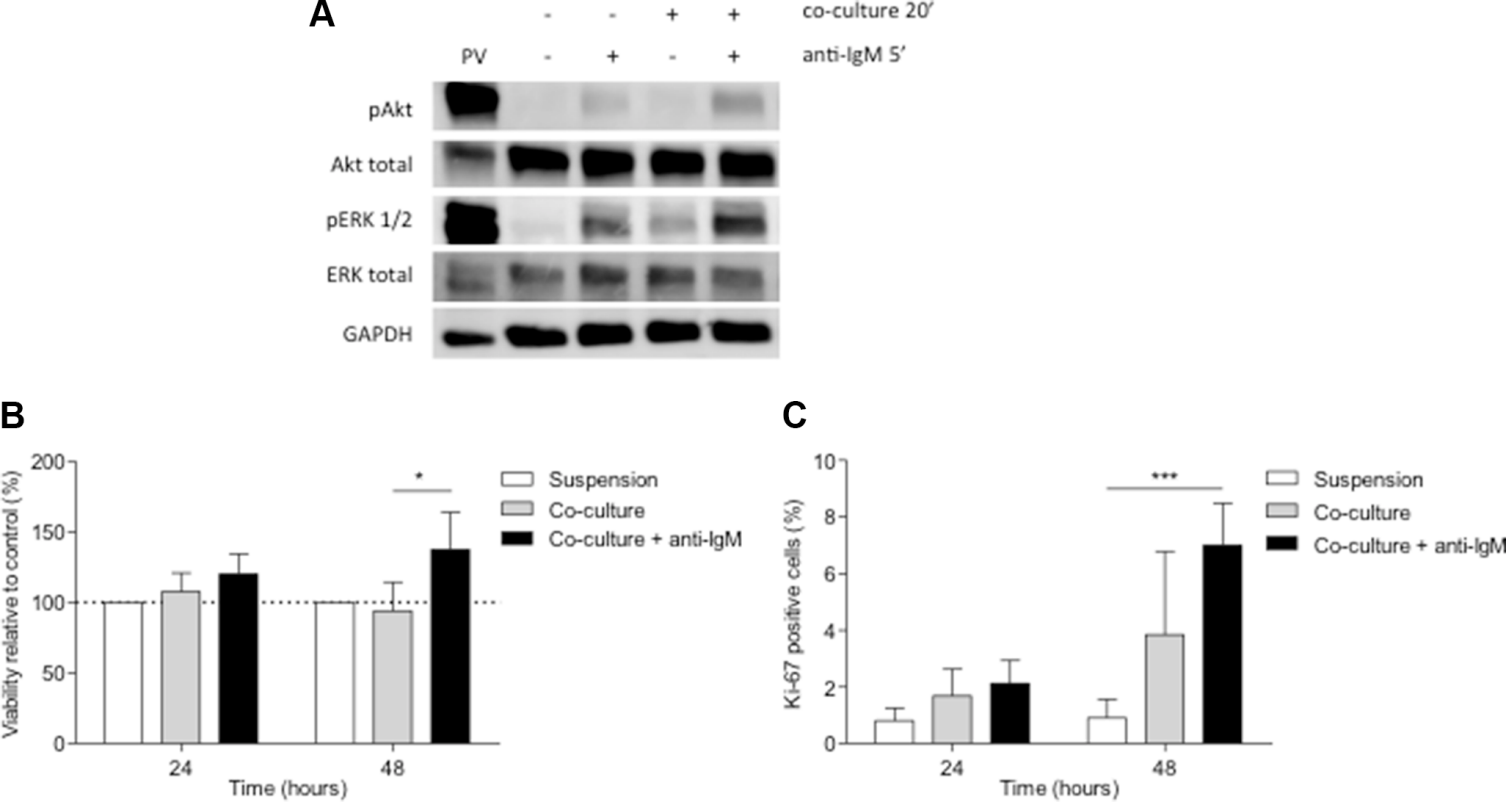
BCR stimulation with anti-IgM increases viability and enhances proliferation in primary CLL cells co-cultured with BMSC, CD40L and CpG ODN (**A**) Primary CLL cells were co-cultured with BMSC, CD40L and CpG ODN for 15 minutes and anti-IgM was added for 1 additional minute. Figure shows the immunoblot analysis of Akt and ERK1/2 phosphorylation from a representative patient. (**B**) Primary CLL cells were co-cultured with BMSC, CD40L, CpG ODN and anti-IgM for 24 and 48 hours. Viability was assessed in primary CLL cells from 9 patients by Annexin V and PI staining. (**C**) Mean % of Ki-67-positive cells from 9 patients was analyzed by FC. (*P < 0.05, ***P < 0.001, two-way ANOVA, Bonferroni's post-test. Graph shows mean ± SEM). PV: treatment with pervanadate.

To *in vitro* reproduce the microenvironment that CLL cells find in the proliferative centers *in vivo*, we co-cultured primary CLL cells with the BMSC cell line UE6E7T-2, soluble CD40L and CpG ODN which induce proliferation and chemoresistance in primary CLL cells as we have recently shown [15]. Western blot analysis showed that phosphorylation of Akt and ERK1/2 after BCR cross-linking was higher in co-cultured CLL cells than in cells in suspension (Figure 1A). Since the activation of BCR pathway induces pro-survival and proliferative signals in CLL cells, [4, 12, 16, 17, 18] we tested the effects of BCR stimulation with anti-IgM in CLL cell viability and proliferation in this co-culture system. We observed that the addition of anti-IgM for 48 hours protected co-cultured CLL cells from undergoing spontaneous apoptosis (Figure 1B) (mean % of viable co-cultured CLL cells related to CLL cells in suspension: 94.08 ± 20.27 without anti-IgM stimulation *vs.* 137.52 ± 26.17 with anti-IgM stimulation, *P* < 0.05). Moreover, proliferative responses were already observed after 24 hours of co-culture although a significant induction of Ki-67 expression was only observed after 48 hours of co-culture with the addition of anti-IgM (Figure 1C) (mean % Ki-67-positive cells: 0.91 ± 0.22 in suspension *vs.* 3.85 ± 0.93 in co-culture, *P* > 0.05, or *vs.* 7.00 ± 1.49 in co-culture with anti-IgM, *P* < 0.001).

### Treatment with TAK-659 inhibits Syk activation and BCR signaling in co-cultured primary CLL cells and Burkitt's lymphoma cells

**Figure 2:**
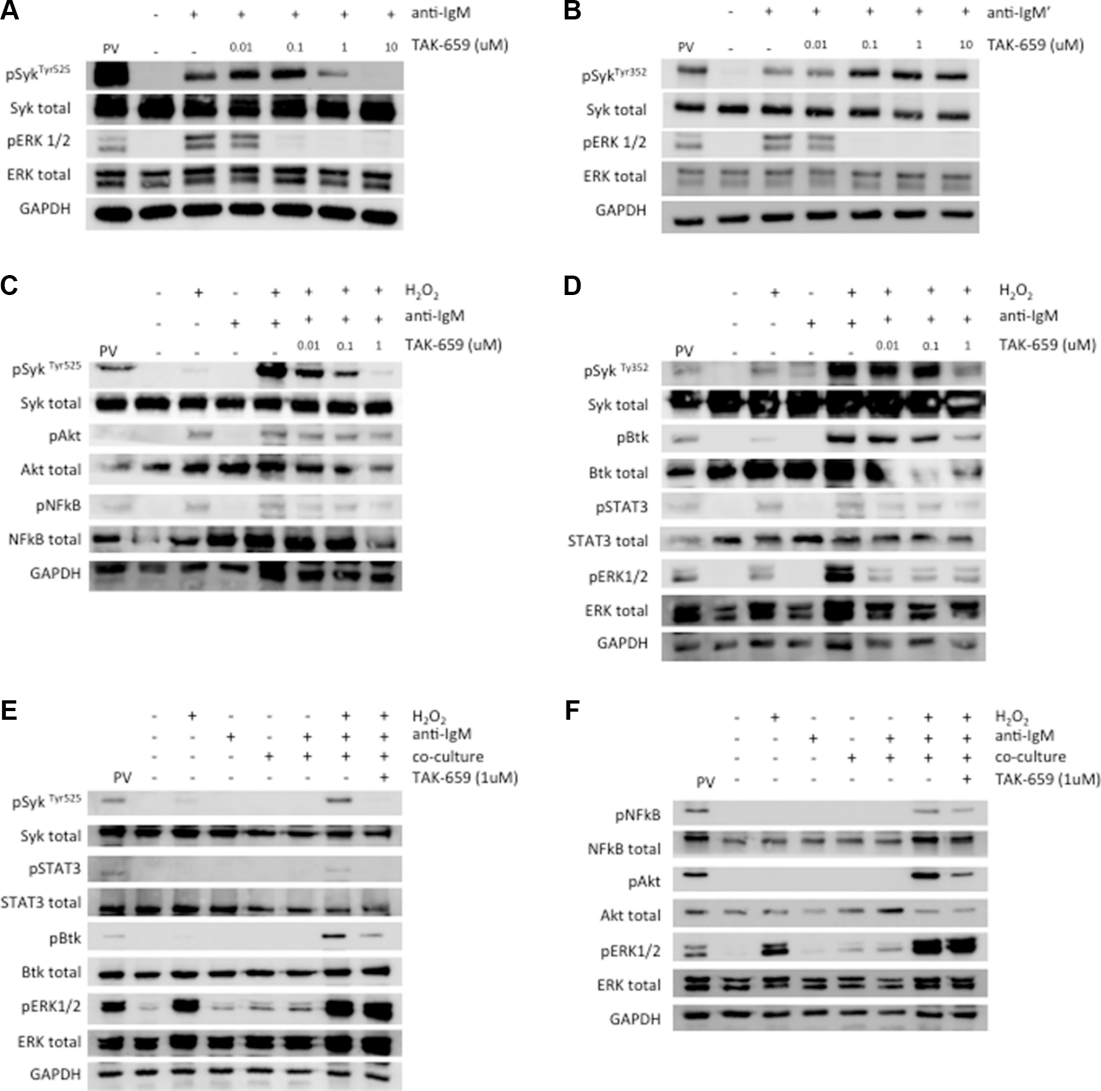
Syk inhibition by TAK-659 downregulates BCR signalling in Ramos and primary CLL cells (**A–B**) Phosphorylation of Syk and ERK1/2 were analyzed by Western Blot in Ramos cells incubated with increasing doses of TAK-659 for 1 hour and stimulated with anti-IgM for 5 minutes. (**C–D**) Phosphorylation of Syk, Btk, NFκB, ERK1/2 and STAT3 were analyzed by Western Blot in primary CLL cells pre-treated with increasing doses of TAK-659 for 1 hour and stimulated with anti-IgM for 5 minutes. (**E–F**) Primary CLL cells pre-treated with increasing doses of TAK-659 for 1 hour were co-cultured with BMSC, CD40L and CpG ODN for 15 minutes and anti-IgM stimulated for additional 4 minutes. H_2_O_2_ 3.3 mM was added for 4 minutes. Expression of phosphorylated Syk, Btk, NFκB, Akt, ERK1/2 and STAT3 was analyzed by western blot. PV: treatment with pervanadate

To determine the effects of the Syk inhibitor TAK-659 on BCR downstream signaling, we firstly used the Burkitt's lymphoma cell line Ramos as a model of mature malignant IgM-positive B-cells. We treated Ramos cells with increasing doses of TAK-659 for 1 hour, and subsequently, we stimulated BCR with anti-IgM for 5 minutes prior to whole protein extraction. Stimulated Ramos cells displayed enhanced expression of phospho-Syk at Tyr525 and Tyr352 and phospho-ERK1/2. Treatment with TAK-659 was able to completely abrogate ERK phosphorylation induced by anti-IgM stimulation. However, we observed that higher doses of TAK-659 were required to completely inhibit phosphorylation of Syk at the TAK-659 binding site, Tyr525, located within the kinase domain. Interestingly, an initial enhancement on phosphorylation of Syk at this site was observed with lower doses of TAK-659. This observation, along with the enhancement on phosphorylation in residue Tyr352 of Syk protein, an activation site within the interdomain B, in response to TAK-659 treatment at any dose, suggest a differential regulation of these sites via a positive feedback (Figure 2A and 2B).

In primary CLL cells in suspension culture, TAK-659 treatment resulted in a dose-dependent reduction in the phosphorylation of Syk^Tyr525^, Btk, NFκB, ERK1/2 and STAT3 after BCR stimulation (Figure 2C and 2D). For robust and consistent detection of phosphorylated proteins, we treated cells with H_2_O_2_, a broad phosphatase inhibitor, as previously described [19]. Finally, in order to assess the effect of TAK-659 in CLL cells being stimulated by different players from the microenvironment we pre-treated CLL cells with TAK-659 1 μM for 1 hour followed by co-culture with BMSC, CD40L and CpG ODN for 15 minutes and subsequent BCR crosslinking for 5 additional minutes. An enhancement on Syk^Tyr525^ was evidenced in co-cultured and BCR-stimulated primary CLL cells with the addition of H_2_O_2_. This Syk^Tyr525^ enhancement was inhibited by TAK-659 (Figure 2E). Phosphorylation of Btk, Akt, NFκB and STAT3 was also decreased after TAK-659 treatment, thus confirming the inhibition of Syk downstream signaling also in this setting. However, contrary to what we observed when cells were only stimulated by anti-IgM, the phosphorylation of ERK1/2 was not inhibited by TAK-659 treatment, reflecting persistent activation of alternative pathways independent from Syk in co-cultured primary CLL cells (Figure 2E and 2F).

### Inhibition of Syk by TAK-659 induces apoptosis of CLL cells and abrogates BCR and co-culture-derived survival signals

**Figure 3:**
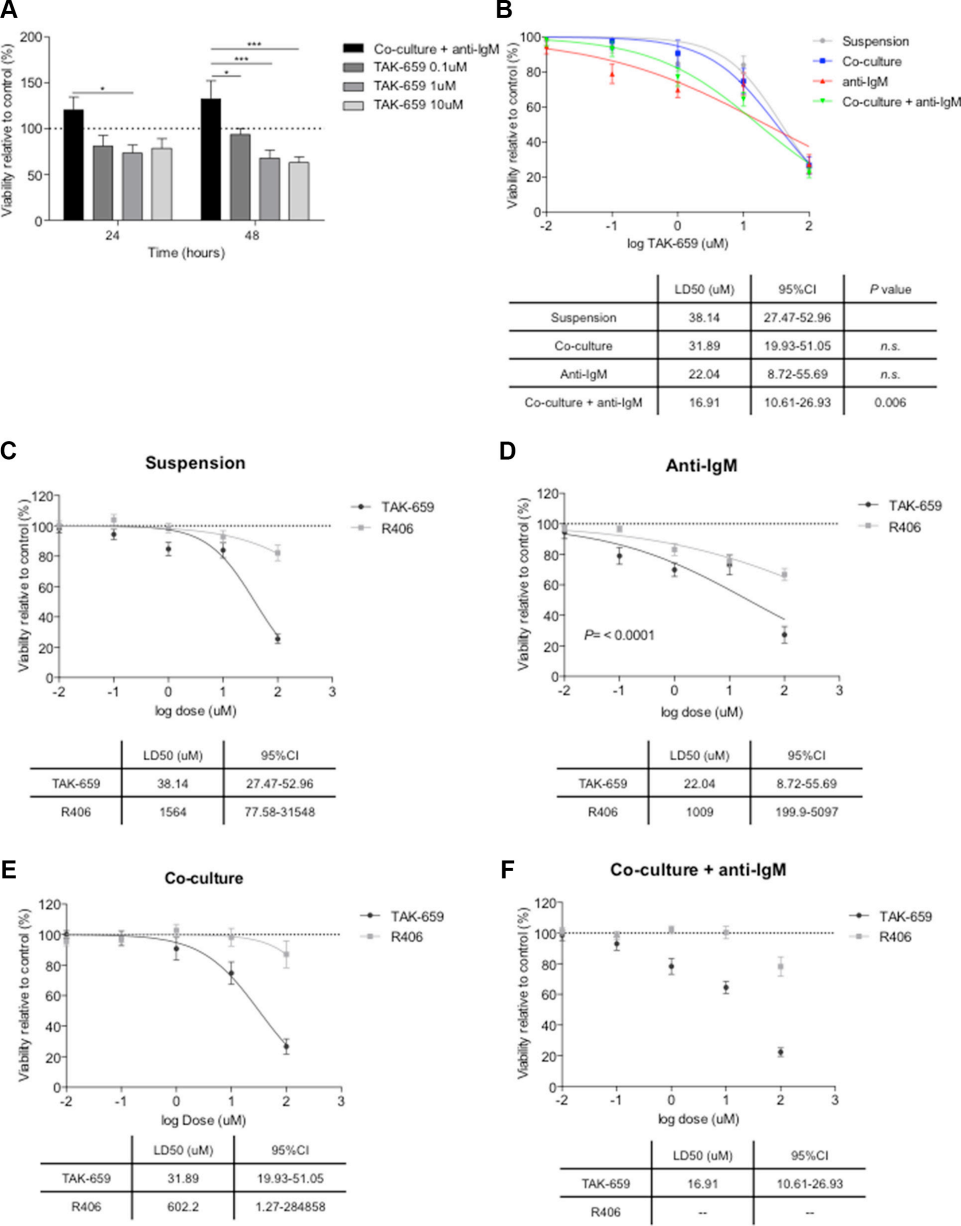
TAK-659 induces higher degree of apoptosis than R406 in primary CLL cells (**A**) Primary CLL cells from 12 patients pre-treated with increasing doses of TAK-659 were cultured for 24 and 48 hours with BMSC, CD40L, CpG ODN and anti-IgM. Viability was assessed by Annexin V and PI staining by FC. Primary CLL cells from 26 patients pre-treated with increasing doses of TAK-659 were cultured for 48 hours in suspension, in co-culture, anti-IgM-stimulated or co-cultured and with anti-IgM stimulation (**B**); or pre-treated with increasing doses of TAK-659 or R406 in suspension (**C**), anti-IgM-stimulated (**D**), in co-culture (**E**) or co-cultured with anti-IgM-stimulation (**F**). LD_50_ curves for TAK-659 and R406 are plotted on a logarithmic scale. LD_50_ for every treatment cohort were calculated and compared (Two-way ANOVA, Bonferroni's post*-test*. Graph shows mean ± SEM).

To determine whether the pro-survival effect of co-culture and BCR stimulation might be abrogated by TAK-659, we treated co-cultured and anti-IgM stimulated CLL cells with increasing doses of TAK-659. As displayed in Figure 3A, TAK-659 significantly reduced the viability of CLL cells in a dose-dependent manner, after 48 hours of treatment (mean % viability relative to untreated CLL cells for TAK-659 0.1 μM, 1 μM and 10 μM after 48 hours: 93.51 ± 6.78 *vs.* 67.91 ± 8.88 *vs.* 63.05 ± 6.39, respectively, *P* < 0.001). Next, to determine differences in the sensitivity to TAK-659 treatment according to the stimuli present in the co-culture system, we cultured primary CLL cells in 4 different conditions: in suspension, stimulated with anti-IgM, co-cultured with BMSC and stimulated with CD40 ligand along with CpG ODN and, co-cultured with the addition of anti-IgM. Increasing doses of TAK-659 were added and viability of CLL cells was assessed after 48 hours of treatment. We observed that the addition of anti-IgM stimulation to the co-culture system provided the greater sensitivity to TAK-659 (TAK-659 LD_50_ for CLL cells in suspension: 38.14 μM [95%CI 27.47–52.96] *vs.* TAK-659 LD_50_ for CLL cells in co-culture and anti-IgM: 16.91 μM [95%CI 10.61–26.93], *P* = 0.006) (Figure 3B). No statistically significant difference was observed between dose effect curves for anti-IgM or co-culture alone compared to CLL cells in suspension although LD_50_ for CLL cells in suspension was higher (TAK-659 LD_50_ for CLL cells with anti-IgM: 22.04 μM [95%CI 8.72–55.69] *vs.* TAK-659 LD_50_ for CLL cells in co-culture: 31.89 μM [95%CI 19.93–51.05], *P* > 0.05) (Figure 3B). We then compared the effects of TAK-659 with those of Syk-inhibitor R406 (fostamatinib) on the viability of primary CLL cells. We observed that TAK-659 resulted clearly more effective than R406 in inducing CLL cells apoptosis in all culture conditions (Figure 3C–3F). TAK-659 displayed a significantly stronger capacity to induce apoptosis in primary CLL cells in suspension, being the LD_50_ for TAK-659 more than 40 times lower than the one observed for R406 (Figure 3C). The stimulation with anti-IgM or the co-culture of primary CLL cells seemed to provide slight higher sensitivity to R406 treatment although not significant. However, the combination of anti-IgM stimulation and co-culture induced a marked resistance to R406 treatment that precluded the calculation of a LD_50_, whereas LD_50_ for TAK-659 was 16.91 μM (95%CI 10.61–26.93 μM) (Figure 3D).

Altogether, this data indicates a strong capacity of TAK-659 to block the microenvironment-derived survival signals and a higher efficacy in co-cultured and BCR-stimulated CLL cells compared to cells in suspension.

### TAK-659 inhibits the up-regulation of proliferation and activation markers induced by the co-culture in primary CLL cells

**Figure 4:**
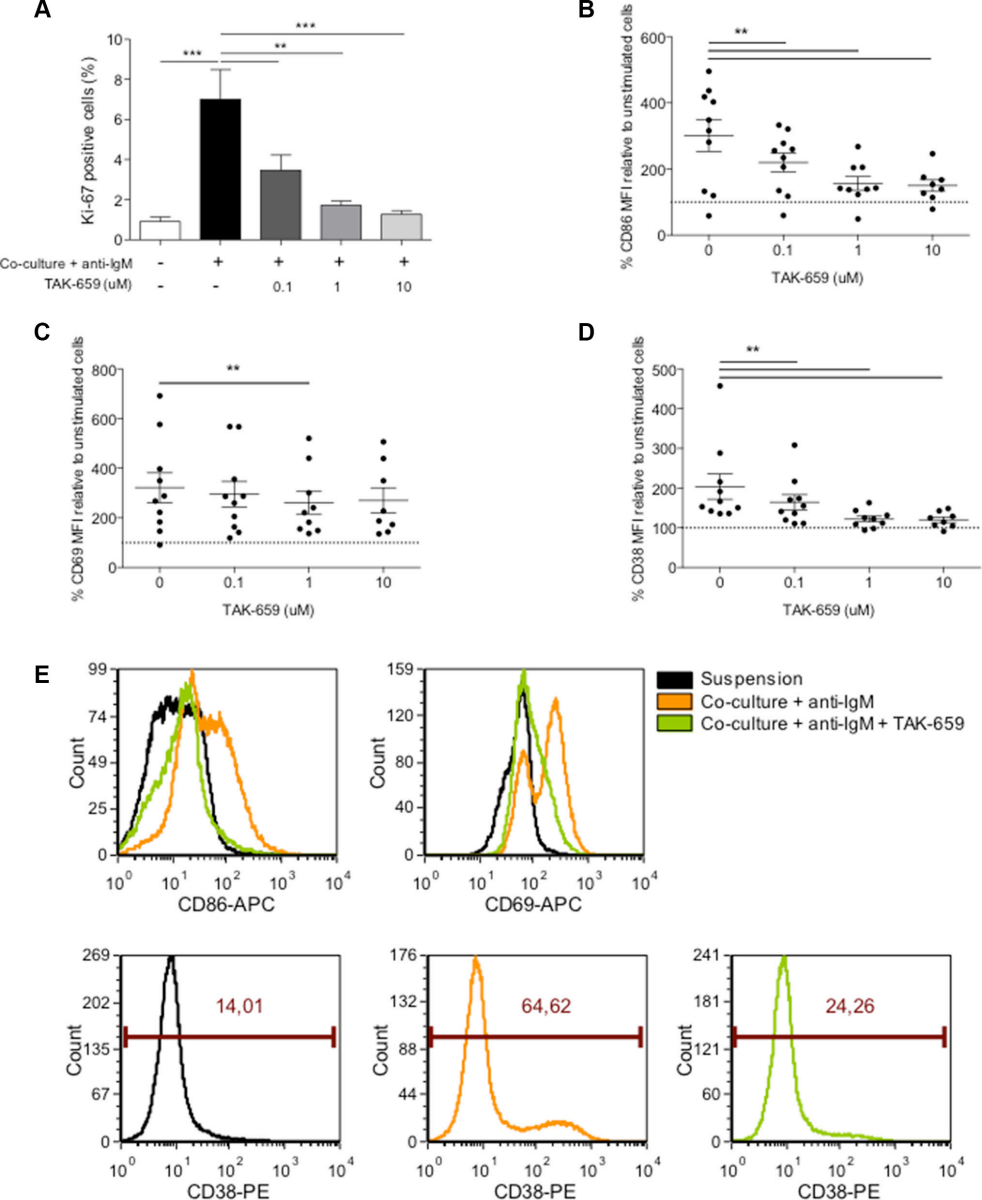
Treatment with TAK-659 effectively abrogates the co-culture-induced proliferation and activation of primary CLL cells Primary CLL cells from 10 patients pre-treated with increasing doses of TAK-659 were cultured with BMSC, CD40L, CpG ODN and anti-IgM for 48 hours and the expression of Ki-67 (**A**), CD86 (**B**), CD69 (**C**) and CD38 (**D**) was analyzed by FC. (**E**) Histograms from a representative patient. (*P < 0.05, **P < 0.01, ***P < 0.001, two-way ANOVA, Bonferroni's post*-test*. Graph shows mean ± SEM).

To test whether TAK-659 was able to override the co-culture-induced proliferation described above (Figure 1C), primary CLL cells were treated with increasing doses of TAK-659 for one hour and subsequently co-cultured for 48 hours. TAK-659 treatment inhibited co-culture-induced proliferation in a dose-dependent manner, being 10 μM the TAK-659 dose at which the percentage of Ki-67 positive CLL cells did not significantly differ from that in unstimulated CLL cells (Figure 4A) (mean % of Ki-67-positive cells: 7.00 ± 1.49 in untreated controls *vs.* 3.39 ± 0.76 in TAK-659 0.1 μM-treated CLL cells, *P* < 0.01 *vs.* 1.72 ± 0.20 in TAK-659 1 μM-treated CLL cells, *P* < 0.01 *vs.* 1.27 ± 0.18 in TAK-659 10 μM-treated CLL cells, *P* < 0.001). We next assessed the effect of TAK-659 on CLL cell activation evaluating expression of CD86, CD69 and CD38. CD86 and CD69 are two activation markers known to be upregulated in CLL cells from LN and BM *in vivo* [4]. CD38 is not only upregulated in activated CLL cells but also serves as prognostic marker [20, 21]. Co-culture of primary CLL cells induced a significant increase in the expression of these activation markers. Interestingly, Syk inhibition by TAK-659 resulted in marked downregulation of the expression of CD86, CD69, and CD38 in a dose-dependent manner though the degree of downregulation of CD69 was smaller compared to that of CD86 or CD38 (Figures 4B, 4C, 4D and 4E).

### TAK-659 inhibits chemotaxis toward BMSC, CXCL12 and CXCL13 in primary CLL cells

**Figure 5:**
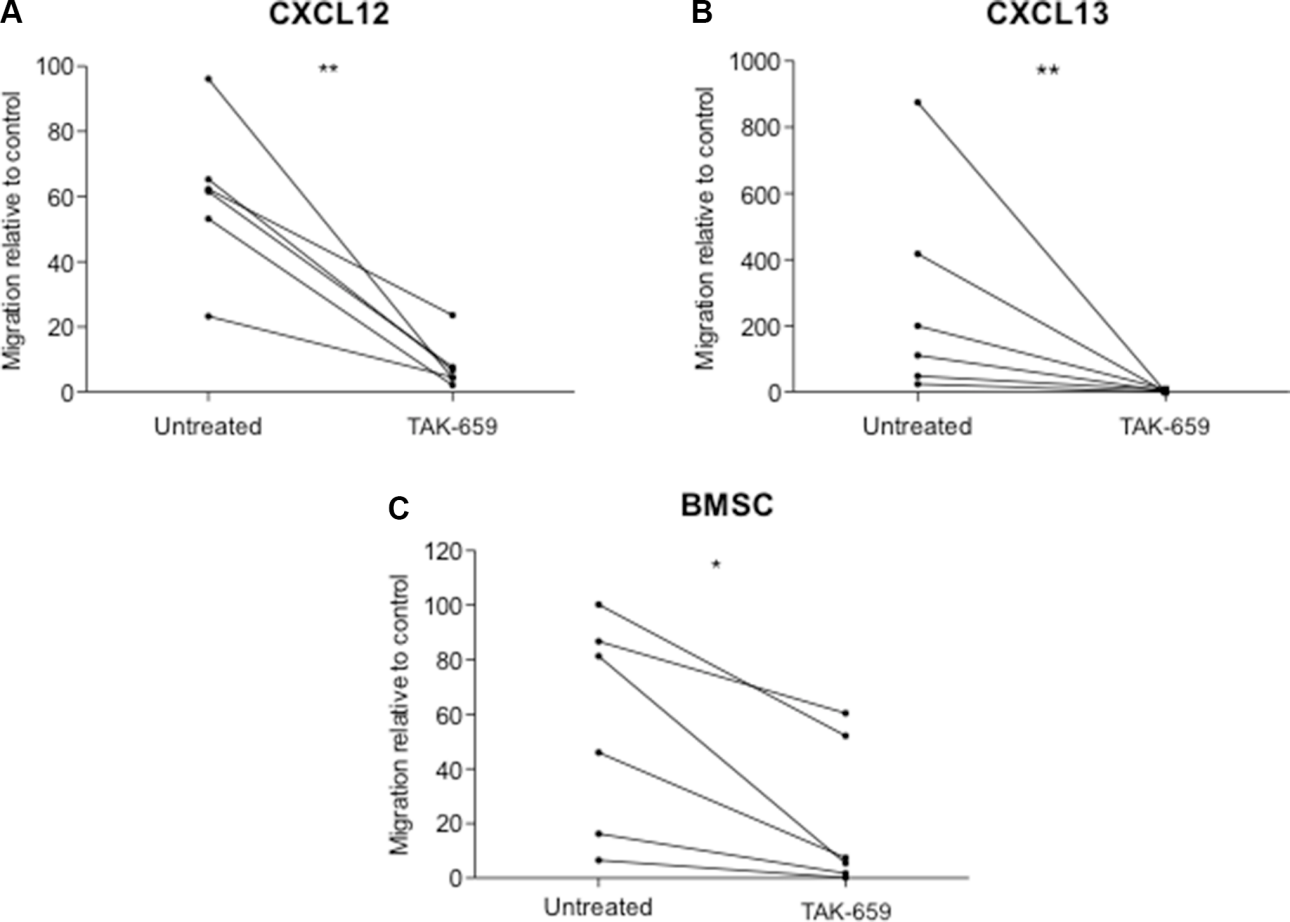
Syk inhibition by TAK-659 inhibits chemotaxis of primary CLL cells toward CXCL12, CXCL13 and BMSC Primary CLL cells from 6 patients were assayed for chemotaxis toward CXCL12, CXCL13 and the BMSC cell line HS-5 for 4 hours after incubation for 1 hour with TAK-659. (*P < 0.05, **P < 0.01, paired *T-test*. Wilcoxon post*-test*).

In addition to survival and proliferative signals, BCR signaling also promotes the homing of CLL cells to the BM and the LN, thus facilitating their access to favorable environments. This is mainly mediated by enhancing the response to CXCR4 and CXCR5 [12, 22, 23, 24]. To evaluate the impact of Syk inhibition by TAK-659 on the *ex vivo* chemotaxis of CLL cells, we analyzed the ability of CLL cells to migrate toward CXCL12, CXCL13 and the BMSC cell line HS-5 by performing transmigration assays using bare polycarbonate membranes. Treatment of primary CLL cells with TAK-659 0.1 μM for 1 hour resulted in a strong reduction of the migratory capacity toward CXCL12, CXCL13 and the BMSC cell line, HS-5 (Figures 5A, 5B and 5C).

### TAK569 abrogates microenvironment-induced chemoresistance

**Figure 6:**
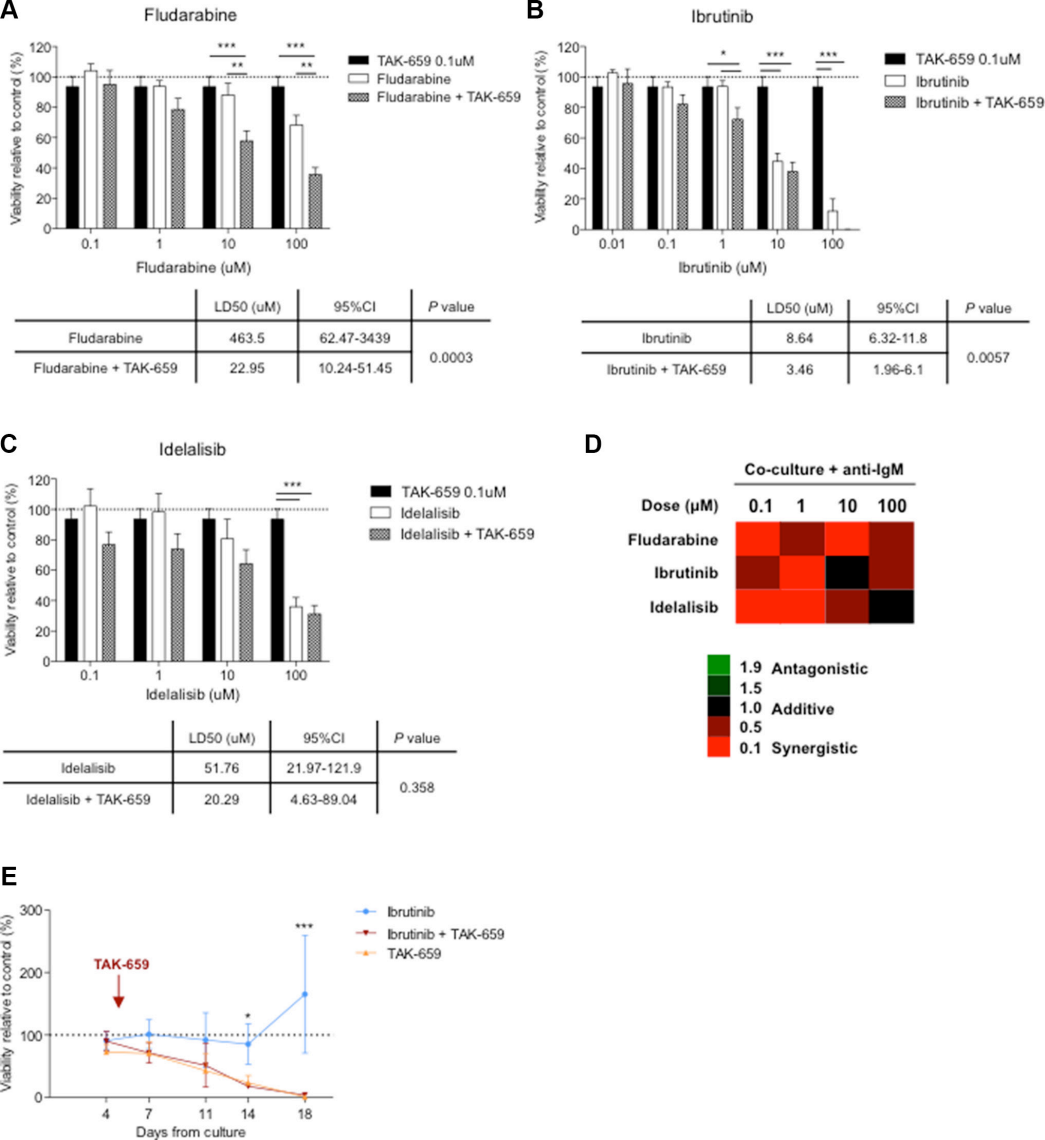
The combination of TAK-659 with fludarabine, ibrutinib or idelalisib synergistically induces apoptosis in proliferative CLL cells Primary CLL cells from 12 patients were pre-treated with 0.1 μM TAK-659 and increasing doses of fludarabine (**A**), ibrutinib (**B**) or idelalisib (**C**) for 1 hour and co-cultured for 48 hours. LD_50_ for every treatment cohort were calculated and compared (*P < 0.05, **P < 0.01, ***P < 0.001, two-way ANOVA, Bonferroni's post -test. Graph shows mean ± SEM). (**D**) Calculated combination index values are plotted. (**E**) Primary CLL cells from 6 patients were pre-treated with 1 μM ibrutinib or 1 μM TAK-659 and cultured with CD40L 1 μg/mL. After 4 days, 1 μM TAK-659 was added to ibrutinib-treated cells. Viability of primary CLL cells was analyzed by Annexin V and PI staining by FC at the indicated time points. (*P < 0.05, ***P < 0.001, paired *T-test*. Wilcoxon post*-test*).

As we previously described, [15, 25] the co-culture of CLL cells with BMSC, CD40L and CpG ODN induces marked resistance to fludarabine and bendamustine in primary CLL cells. To test whether the addition of TAK-659 might overcome this co-culture-induced chemoresistance, we assessed the effects of the combination of TAK-659 with fludarabine. For that purpose, we compared the viability rates after treatment with increasing doses of fludarabine with or without 0.1 μM TAK-659 in primary CLL cells co-cultured with BMSC, CD40L and CpG ODN with the addition of anti-IgM. We chose this dose of TAK-659 according to the significant effects in terms of proliferation, activation, migration and inhibition of BCR signaling mentioned before. In line with our previous observations, [15] this co-culture system inhibited fludarabine-induced apoptosis in primary CLL cells. Interestingly, we observed that this co-culture-induced chemoresistance was markedly reverted by the addition of TAK-659 (Figure 6A). Subsequent calculation of the cooperative index between the two drugs at different fludarabine concentrations indicates that simultaneous treatment with TAK-659 and fludarabine synergistically induces apoptosis in co-cultured CLL cells independently from fludarabine dose (Figure 6D).

### TAK-659 synergizes with other BCR inhibitors in co-cultured CLL cells

To investigate the effects of the combination of TAK-659 with other BCR inhibitors we treated primary CLL cells with 0.1 μM TAK-659 combined with increasing doses of the Btk inhibitor, ibrutinib, and the PI3K delta-specific isoform inhibitor, idelalisib, and compared their viability rates. We observed that apoptosis was significantly increased by TAK-659 in ibrutinib-treated co-cultured CLL cells (Figure 6B). Interestingly, cooperative index values revealed that TAK-659 strongly potentiated ibrutinib cytotoxicity especially at 1μM of ibrutinib in co-cultured CLL cells (Figure 6D). Although we observed no significant difference in the induction of apoptosis between treatment with idelalisib as single agent and in combination with TAK-659 (Figure 6C), synergic cytotoxicity was also demonstrated with the combination. Similarly to the combination of TAK-659 with ibrutinib, the combination with idelalisib displayed highest synergistic effect (lowest cooperative values) at the lowest idelalisib dose tested (Figure 6D).

### Sequential treatment with TAK-659 strongly enhances long-term cytotoxicity in ibrutinib-treated CLL cells

Despite the incontestable success in the treatment of CLL shown so far, a progression rate of 5.3% has been already reported in patients under ibrutinib treatment [7, 8]. Resistance to ibrutinib has been related to the overgrowth of subclones with BTK or PLCg2 mutations, resulting in reactivation of the BCR pathway [26]. To test whether the upstream inhibition of BCR signaling with TAK-659 could have a cytotoxic effect in cells that have been under the pressure of ibrutinib treatment we designed an experimental approach using a long-term culture system with CD40-stimulated primary CLL cells. In that culture system we treated CLL cells with ibrutinib for 4 days and subsequently, TAK-659 was added. We used CLL cells treated with either ibrutinib or TAK-659 alone as control. Interestingly, we observed that CD40-stimulated primary CLL cells persisted insensitive, in terms of cytoxicity, to 1 μM ibrutinib treatment along time. By contrast, sequential treatment with TAK-659 efficiently decreased the viability of these *‘ibrutinib-resistant*’ primary CLL cells (mean % viability relative to untreated CLL cells for ibrutinib, TAK-659 or ibrutinib and TAK-659 after 14 days: 85.45 ± 32.10 *vs.* 23.61 ± 11.62 *vs.* 17.89 ± 1.43, respectively, *P* < 0.05; and after 18 days: 165.22 ± 94.10 *vs.* 0.89 ± 0.01 *vs.* 3.92 ± 3.20, respectively, *P* < 0.001) (Figure 6E).

### TAK-659 does not inhibit TCR signaling and molecular features of T cell activation in primary T cells from patients with CLL

The primary aminoacid sequence of Syk is homologous to that of ZAP-70, the expression of which is mostly confined to T and natural killer (NK) cells [27]. Given the striking homology between these two Syk family kinases we hypothesized that TAK-659 might be a potential ZAP-70 inhibitor. This might have crucial consequences such as suppression of T cell-based immune responses or antibody-dependent NK-cell mediated cytotoxicity (ADCC), as shown for ibrutinib inhibiting Itk protein which has sequence and functional homology to Btk protein [28, 29].

**Figure 7:**
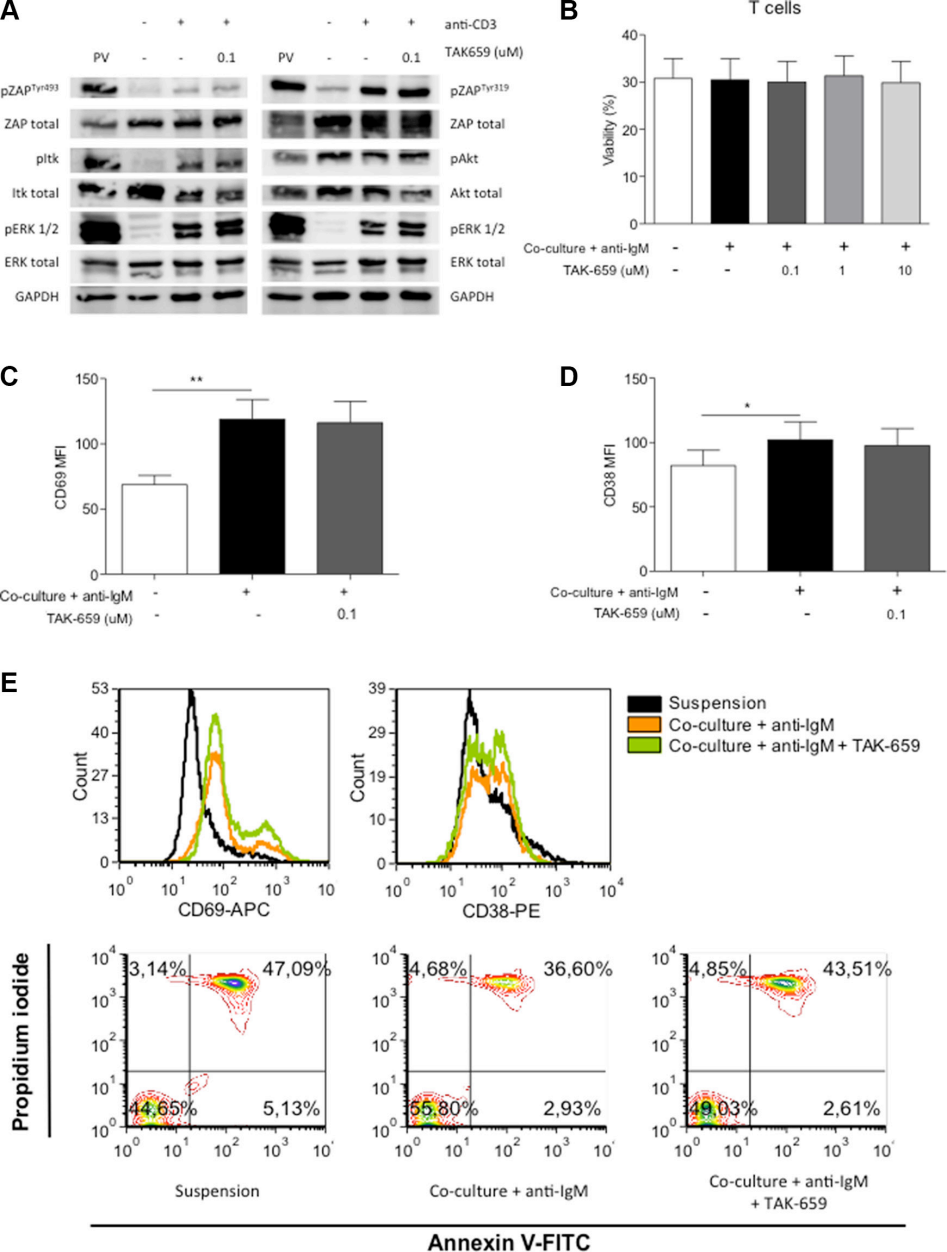
TAK-659 does not inhibit TCR downstream signaling or expression of activation molecules in T cells (**A**) Jurkat cells were incubated with 0.1 μM TAK-659 for 1 hour and stimulated with anti-CD3 for 1 minute. Expression of phospho-proteins was analyzed by western blot. Jurkat cells treated with pervanadate (PV) were used as positive controls. (**B**) PBMC from 10 patients diagnosed with CLL were incubated with increasing doses of TAK-659 for 1 hour and cultured in suspension or in co-culture with BMSC, CD40L, CpG ODN and anti-IgM for 48 hours. Viability of T cells was assessed by Annexin V and PI staining in CD3^+^ cells by FC. (**C–D**) PBMC from 10 patients diagnosed with CLL were incubated with 0.1 μM TAK-659 for 1 hour and cultured in suspension or in co-culture with BMSC, CD40L, CpG ODN and anti-IgM for 48 hours. The expression levels of CD69 and CD38 were analyzed in CD19^−^/CD3^+^ cells by FC. (**E**) Histograms for the expression of CD69 and CD38 along with dot-plots for the expression of Annexin V and PI in CLL cells cultured in suspension or in co-culture with anti-IgM with or without TAK-659 from a representative patient are shown. (*P < 0.05, **P < 0.01, paired *T-test*, Wilcoxon post-*test*. Graph shows mean ± SEM).

To investigate the effects of TAK-659 on T cells, we firstly determined the effects on anti-CD3-induced TCR signalling in Jurkat T cells. Immunoblot data revealed that phosphorylation of ZAP-70^Tyr319^, ZAP-70^Tyr493^, Itk, Akt and ERK1/2 were not affected by the treatment with TAK-659 (Figure 7A). Further, we evaluated the apoptosis rate in the CD3+ cells obtained from CLL patients; these cells were cultured in our co-culture system and pre-treated with increasing doses of TAK-659. In contrast to primary CLL cells, we found that the co-culture did not modify the viability of primary T cells and that the addition of TAK-659 did not induce apoptosis in these cells (Figure 7B). We also analyzed the effects of TAK-659 on T cell activation by determining the expression of the activation markers CD69 and CD38 by FC. We observed that culturing T cells in our co-culture system induced the expression of both molecules and that this upregulation was not modified after TAK-659 treatment (Figures 7C, 7D and 7E).

## DISCUSSION

Successful targeting of tyrosine kinases that are crucial for BCR signaling has been one of the major recent breakthroughs in the treatment of patients with B-cell malignancies, particularly CLL. Syk protein has a crucial role in transmitting signaling from the BCR and from other receptors, such as CXCR4, providing it with an outstanding relevance as a therapeutic target for CLL [10, 11]. In this sense, targeting Syk has been previously explored using fostamatinib, [11, 10, 12, 30] although some activity was reported in a phase 2 study in NHL and CLL, [13] further clinical development in B-cell malignancies has not been reported. Recently, entospletinib (GS-9973), has been, demonstrated to have clinical activity and acceptable toxicity in patients with relapsed or refractory CLL [31]. TAK-659, a new Syk inhibitor, has recently shown antitumor activity in lymphoma xenograft models [32] and in diffuse large B cell lymphoma patients according to preliminary data from the first-in-human phase 1 clinical trial [33].

In the present study, TAK-659 was able to completely block the anti-IgM-induced phosphorylation of Btk, NFκB and ERK1/2. Of note, TAK-659 treatment also led to the inhibition of STAT3^Tyr705^ phosphorylation, as recently described with fostamatinib [34]. Moreover, we observed persistence of phosphorylation of Syk^Tyr352^ and ERK1/2 after TAK-659 treatment, suggesting the existence of molecular feedback loops that would activate Syk^Tyr352^ and alternative signaling pathways, such as CXCR4, responsible for ERK1/2 activation when BCR signaling is inhibited. In this regard, Syk^Tyr352^ phosphorylation through Lyn activation in a feedback response to Syk inhibition by fostamatinib has been previously described [35].

Clinically, BCR inhibitors cause CLL cell redistribution from tissues into the PB [7, 36]. Reduced CLL cell adhesion to stromal cells might promote their mobilization to the PB where a reduced responsiveness to CXCL12 and CXCL13 might block CLL cell tissue homing. In this report we have demonstrated that TAK-659 is able to effectively impair CLL cell migration toward CXCL12, CXCL13 and the BMSC cell line HS-5, in line with previous studies with other BCR inhibitors [37, 38].

Herein, we have also shown how TAK-659 is able to efficiently induce apoptosis in primary CLL cells preferentially in proliferative culture conditions. As seen with other kinase inhibitors like ibrutinib or idelalisib, [37–40] TAK-659 requires doses at the micromolar range to achieve significant cytotoxicity in CLL cells despite its inhibitory dose 50 for enzymatic activity is 3.2 nM [32].

The diverse signaling from the microenvironment is also involved in the induction of resistance to fludarabine. Herein, we show that TAK-659 is able to overcome this microenvironment-induced resistance to fludarabine, and that the combination with ibrutinib or idelalisib synergistically decreased CLL cell viability. Significant induction of apoptosis was specifically seen in co-cultured CLL cells suggesting that the difficulty in eradicating this CLL cell compartment might be successfully targeted with combination treatment strategies. In this sense, cross-talk between signaling pathways and feedback loops induced by continuous inhibition of a single target might drive resistance and have impact on the efficacy of single agent therapy. Therefore, we foresee a near future where the simultaneous or sequential inhibition of multiple CLL cell signaling pathways might progressively become relevant for overcoming resistance to single agents. Further, our experiments with extended culture of primary CLL cells treated with ibrutinib, TAK-659 or the sequential combination of both drugs suggest that combinations of different BCR signaling inhibitors, may be an efficient therapeutical approach. Recently, ibrutinib has been shown to irreversibly bind to and inhibit the Btk homologue Itk protein, therefore inhibiting Th2 activation after TCR stimulation. Moreover, Itk inhibition by ibrutinib also led to antagonize anti-CD20 antibodies-dependent NK-cell mediated cytotoxicity, therefore having a negative interaction that should be considered when designing combination therapies including these two types of drugs. Similar effects have been observed with idelalisib [28, 41, 29]. In contrast, we showed that TAK-659 treatment does not inhibit the Syk homologue ZAP-70 since downregulation of TCR-derived signaling or inhibition of T cell activation were not observed. These results are in agreement with previous kinase activity assays that showed that TAK-659 IC_50_ for inhibiting ZAP-70 was 87 nM compared to 3.2 nM for inhibiting Syk [32].

In conclusion, our work contributes to the establishment of Syk inhibition using TAK-659 as a rational therapeutic strategy for patients with CLL.

## MATERIALS AND METHODS

### Isolation and culture of primary CLL cells

Peripheral blood mononuclear cells (PBMC) from 26 patients diagnosed with CLL were isolated by Ficoll-Paque Plus (GE Healthcare, Buckinghamshire, United Kingdom) density gradient and stored in liquid nitrogen until analysis. Only samples with ≥ 85% of CD19+/CD5+ cells CLL cells, as assessed by flow cytometry (FC), were included in the study. A written informed consent was obtained from all patients in accordance with the Declaration of Helsinki, and the study was approved by the local ethical committee.

### Cell lines

The UE6E7T-2 and HS-5 human bone marrow stromal cells (BMSC) cells line were obtained from Riken Cell Bank (Ibaraki, Japan) and ATCC (Manassas, VA, USA), respectively. Cells were cultured at 37°C in 5% CO_2_ atmosphere in Dulbecco's Modified Eagle Medium (DMEM; Gibco, Carbbad, CA, USA) supplemented with 2 mM L-glutamine, 10% heat-inactivated fetal bovine serum (FBS) and 50 μg/mL penicillin/streptomycin. The T-cell acute lymphoblastic leukemia cell line Jurkat and the Burkitt's lymphoma cell line Ramos were obtained from ATCC and were cultured in RPMI 1640 medium supplemented with 2 mM L-glutamine, 10% heat-inactivated FBS and 50 μg/mL penicillin/streptomycin. All cell lines were obtained more than 6 months before the date of the experiments, and they were not authenticated by the authors.

### Co-culture conditions

Following the method we previously described, [25] BMSCs were seeded at a concentration of 1.5 × 10^4^ cells/mL in 24-well plates and incubated for 24 hours to allow cells to adhere. CLL cells were cultured at a ratio of 100:1 (1.5×10^6^cells/mL) on confluent layers of BMSC in RPMI-10%FCS supplemented with 1 μg/mL CD40L (Peprotech, London, United Kingdom) and 1.5 μg/mL CpG ODN (ODN2006; Invivogen, San Diego, CA, USA). Cross-linking of BCR on primary CLL cells and Ramos cells was performed with 10 μg/mL goat anti-human IgM F(ab')_2_ fragment (Invitrogen, Carlsbad, CA, USA). At the indicated time points, CLL cells were harvested by gently washing off, leaving the adherent stromal cell layer intact.

### Flow cytometry

Intracellular staining of Ki-67 was performed using a fluorescein isothiocyanate (FITC)-labeled antibody against Ki-67 (Becton Dickinson, Franklin Lakes, NJ, USA) after fixation and permeabilization using the BD Intrasure kit (Becton Dickinson) following the manufacturer's instructions. Surface staining of cells was performed using the following monoclonal antibodies conjugated with the indicated fluorochrome: CD19-phycoerythrin (PE) and CD5-allophycocyanine (APC) (Becton Dickinson).

Expression of CD69, CD38 and CD86 in CD19^+^/CD5^+^ and CD3^+^ cells was assessed using the following antibodies: CD19-energy coupled dye (ECD), CD5-phycoerythrincyanine 5.5 (PC5.5) (Beckman Coulter, Brea, CA, USA), CD38-PE (EBioscience, San Diego, CA, USA), CD3-PE-cyanine 7 (Cy7), CD69-APC and CD86-APC (Becton Dickinson). Cells were acquired in a Navios^TM^ cytometer (Beckman Coulter, Brea, CA, USA) and the results were analyzed using the FCS Express 4 software (De Novo Software, Los Angeles, CA, USA).

### Western blot analysis

Ramos, Jurkat and primary CLL cells treated with the phosphatase-inhibitor pervanadate (3 mM H_2_O_2_/1 mM NaVO_4_) for 5 minutes or 3.3 mM H_2_O_2_ for 4 minutes at 37°C were used as positive controls for phospho-proteins. Whole cell protein extracts were prepared from 10 × 10^6^ cells using 50 μL lysis buffer containing 20 mM Tris(hydroxymethyl)aminomethane (Tris) pH 7.4, 1 mM EDTA, 140 mM NaCl, 1% NP-40 supplemented with 2 mM sodium vanadate and protease inhibitor cocktail (Sigma-Aldrich, San Louis, MO, USA) for 1 hour at 4°C. Protein concentration was determined using the Bio-Rad protein assay (Bio-Rad, Hercules, CA, USA). Equal amounts of denatured protein were resolved by 10% SDS-PAGE and transferred to Immobilon-P membranes (Millipore, Bedford, MA, USA). Membranes were blocked for 1 hour at room temperature (RT) in 3% milk/TBS-T. Membranes were incubated overnight at 4°C with primary antibodies against phospho-Syk^Tyr352^/phosho-ZAP-70^Tyr319^, phospho-ZAP-70^Tyr493^, phospho-Akt^Ser473^, phospho-ERK1/2^Thr202/Tyr204^, phospho-STAT3^Tyr705^, Akt, ERK1/2 and STAT3 (Cell Signaling Technology), phospho-NFκB p65^Ser536^, Syk and ZAP-70 (Santa Cruz Biotechnologies, Dallas, TX, USA), phospho-Btk^Tyr551^/phospho-Itk^Tyr511^, Btk and Itk (BD Biosciences), phospho-Syk^Tyr525^ and GAPDH (Abcam, Cambridge, United Kingdom), and NFκB p65 (Chemicon, Millipore). Immunodetection was done with the corresponding IgG HRP-linked secondary antibodies (Dako North America, Glostrup, Denmark), and the ECL chemiluminescence detection system (GE Healthcare).

### Reagents

TAK-659 (kindly provided by Takeda Pharmaceutical International Co.), fludarabine (Sigma), ibrutinib, idelalisib and R-406 (Selleckchem, Houston, TX, USA) were dissolved in DMSO and stored at −20°C.

### Assessment of apoptosis

Apoptosis was assessed analyzing the binding of annexin V-FITC and the incorporation of propidium iodide (PI) by FC. Annexin V/PI double negative cells were considered viable cells. Staining was performed according to the manufacturer's instructions using the annexin V-FITC apoptosis detection kit (Bender Medsystems, Vienna, Austria).

### Chemotaxis assays

Migration toward the chemokines CXCL12 and CXCL13, and to the human BMSC cell line HS-5 was determined in primary cells from 6 patients with CLL by using a transwell migration assay across bare polycarbonate membranes (Corning Incorporated, Corning, NY, USA). A total of 100 μL of RPMI-0.5% bovine serum albumin (BSA) containing 1.5×10^6^ CLL cells were pre-incubated for 1 hour with TAK-659 before they were added to the top chamber of a 6.5-mm-diameter transwell culture insert with a pore size of 5 μm. Inserts were then transferred into wells containing 600 μL RPMI-0.5% BSA with or without 200 ng/mL CXCL12 or 1 μg/mL CXCL13. Cells were allowed to migrate toward the lower chamber for 4 hours at 37°C in 5% CO_2_. To determine migration of CLL cells toward BMSCs, 1.5 × 10^4^ HS-5 cells were seeded and cultured overnight with 600 μL DMEM-10% FBS on the lower chamber. CLL cells were allowed to migrate for 24 hours at 37°C in 5% CO_2_. The number of CLL cells in lower chambers was then determined by FC. The migration index was calculated as the number of CLL cells transmigrating with chemokine or stromal cells divided by the number of transmigrating cells with control medium only.

### Statistical analysis

Results are expressed as the mean ± standard error of the mean (SEM) of at least three independent experiments. The statistically significant difference between groups was analyzed using the Mann-Whitney test or one or two-way ANOVA (*t* test), and *P* < 0.05 was considered significant. Lethal dose 50 (LD_50_) values were calculated with GraphPad Prism software version 5.0 (San Diego, CA, USA). Chou-Talalay method was used for synergy quantification [42]. Analyses were performed using the biostatistics software package SPSS version 22 (IBM, Chicago, IL, USA). Results were graphed with GraphPad Prism software.

## References

[1] Chiorazzi N, Rai KR, Ferrarini M (2005). Chronic lymphocytic leukemia. N Engl J Med.

[2] Burger JA, Tsukada N, Burger M, Zvaifler NJ, Dell'Aquila M, Kipps TJ (2000). Blood-derived nurse-like cells protect chronic lymphocytic leukemia B cells from spontaneous apoptosis through stromal cell-derived factor-1. Blood.

[3] Stevenson FK, Krysov S, Davies AJ, Steele AJ, Packham G (2011). B-cell receptor signaling in chronic lymphocytic leukemia. Blood.

[4] Herishanu Y, Pérez-Galán P, Liu D, Biancotto A, Pittaluga S, Vire B, Gibellini F, Njuguna N, Lee E, Stennett L, Raghavachari N, Liu P, McCoy JP (2011). The lymph node microenvironment promotes B-cell receptor signaling, NF-kappaB activation, and tumor proliferation in chronic lymphocytic leukemia. Blood.

[5] Chen L, Widhopf G, Huynh L, Rassenti L, Rai KR, Weiss A, Kipps TJ (2002). Expression of ZAP-70 is associated with increased B-cell receptor signaling in chronic lymphocytic leukemia. Blood.

[6] Richardson SJ, Matthews C, Catherwood MA, Alexander HD, Carey BS, Farrugia J, Gardiner A, Mould S, Oscier D, Copplestone JA, Prentice AG (2006). ZAP-70 expression is associated with enhanced ability to respond to migratory and survival signals in B-cell chronic lymphocytic leukemia (B-CLL). Blood.

[7] Byrd JC, Furman RR, Coutre SE, Flinn IW, Burger JA, Blum KA, Grant B, Sharman JP, Coleman M, Wierda WG, Jones JA, Zhao W, Heerema NA (2013). Targeting BTK with ibrutinib in relapsed chronic lymphocytic leukemia. N Engl J Med.

[8] O'Brien S, Furman RR, Coutre SE, Sharman JP, Burger JA, Blum KA, Grant B, Richards DA, Coleman M, Wierda WG, Jones JA, Zhao W, Heerema NA (2014). Ibrutinib as initial therapy for elderly patients with chronic lymphocytic leukaemia or small lymphocytic lymphoma: an open-label, multicentre, phase 1b/2 trial. Lancet Oncol.

[9] Sada K, Takano T, Yanagi S, Yamamura H (2001). Structure and function of Syk protein-tyrosine kinase. J Biochem (Tokyo).

[10] Buchner M, Fuchs S, Prinz G, Pfeifer D, Bartholomé K, Burger M, Chevalier N, Vallat L, Timmer J, Gribben JG, Jumaa H, Veelken H, Dierks C (2009). Spleen tyrosine kinase is overexpressed and represents a potential therapeutic target in chronic lymphocytic leukemia. Cancer Res.

[11] Gobessi S, Laurenti L, Longo PG, Carsetti L, Berno V, Sica S, Leone G, Efremov DG (2009). Inhibition of constitutive and BCR-induced Syk activation downregulates Mcl-1 and induces apoptosis in chronic lymphocytic leukemia B cells. Leukemia.

[12] Quiroga MP, Balakrishnan K, Kurtova AV, Sivina M, Keating MJ, Wierda WG, Gandhi V, Burger JA (2009). B-cell antigen receptor signaling enhances chronic lymphocytic leukemia cell migration and survival: specific targeting with a novel spleen tyrosine kinase inhibitor, R406. Blood.

[13] Friedberg JW, Sharman J, Sweetenham J, Johnston PB, Vose JM, LaCasce A, Schaefer-Cutillo J, De Vos S, Sinha R, Leonard JP, Cripe LD, Gregory SA, Sterba MP (2010). Inhibition of Syk with fostamatinib disodium has significant clinical activity in non-Hodgkin lymphoma and chronic lymphocytic leukemia. Blood.

[14] Herman SEM, Barr PM, McAuley EM, Liu D, Wiestner A, Friedberg JW (2013). Fostamatinib inhibits B-cell receptor signaling, cellular activation and tumor proliferation in patients with relapsed and refractory chronic lymphocytic leukemia. Leukemia.

[15] Purroy N, Abrisqueta P, Carabia J, Carpio C, Palacio C, Bosch F, Crespo M (2014). Co-culture of primary CLL cells with bone marrow mesenchymal cells, CD40 ligand and CpG ODN promotes proliferation of chemoresistant CLL cells phenotypically comparable to those proliferating *in vivo*. Oncotarget.

[16] Petlickovski A, Laurenti L, Li X, Marietti S, Chiusolo P, Sica S, Leone G, Efremov DG (2005). Sustained signaling through the B-cell receptor induces Mcl-1 and promotes survival of chronic lymphocytic leukemia B cells. Blood.

[17] Krysov S, Dias S, Paterson A, Mockridge CI, Potter KN, Smith K-A, Ashton-Key M, Stevenson FK, Packham G (2012). Surface IgM stimulation induces MEK1/2-dependent MYC expression in chronic lymphocytic leukemia cells. Blood.

[18] Longo PG, Laurenti L, Gobessi S, Petlickovski A, Pelosi M, Chiusolo P, Sica S, Leone G, Efremov DG (2007). The Akt signaling pathway determines the different proliferative capacity of chronic lymphocytic leukemia B-cells from patients with progressive and stable disease. Leukemia.

[19] Palomba ML, Piersanti K, Ziegler CGK, Decker H, Cotari JW, Bantilan K, Rijo I, Gardner JR, Heaney M, Bemis D, Balderas R, Malek SN, Seymour E (2014). Multidimensional single-cell analysis of BCR signaling reveals proximal activation defect as a hallmark of chronic lymphocytic leukemia B cells. PloS One.

[20] Damle RN, Temburni S, Calissano C, Yancopoulos S, Banapour T, Sison C, Allen SL, Rai KR, Chiorazzi N (2007). CD38 expression labels an activated subset within chronic lymphocytic leukemia clones enriched in proliferating B cells. Blood.

[21] Rassenti LZ, Jain S, Keating MJ, Wierda WG, Grever MR, Byrd JC, Kay NE, Brown JR, Gribben JG, Neuberg DS, He F, Greaves AW, Rai KR (2008). Relative value of ZAP-70, CD38, and immunoglobulin mutation status in predicting aggressive disease in chronic lymphocytic leukemia. Blood.

[22] Bleul CC, Fuhlbrigge RC, Casasnovas JM, Aiuti A, Springer TA (1996). A highly efficacious lymphocyte chemoattractant, stromal cell-derived factor 1 (SDF-1). J Exp Med.

[23] Burger JA, Burger M, Kipps TJ (1999). Chronic Lymphocytic Leukemia B Cells Express Functional CXCR4 Chemokine Receptors That Mediate Spontaneous Migration Beneath Bone Marrow Stromal Cells. Blood.

[24] Ohl L, Henning G, Krautwald S, Lipp M, Hardtke S, Bernhardt G, Pabst O, Förster R (2003). Cooperating mechanisms of CXCR5 and CCR7 in development and organization of secondary lymphoid organs. J Exp Med.

[25] Purroy N, Abrisqueta P, Carabia J, Carpio C, Calpe E, Palacio C, Castellví J, Crespo M, Bosch F (2014). Targeting the proliferative and chemoresistant compartment in chronic lymphocytic leukemia by inhibiting survivin protein. Leukemia.

[26] Cheng S, Guo A, Lu P, Ma J, Coleman M, Wang YL (2015). Functional characterization of BTK(C481S) mutation that confers ibrutinib resistance: exploration of alternative kinase inhibitors. Leukemia.

[27] Chan AC, Iwashima M, Turck CW, Weiss A (1992). ZAP-70: a 70 kd protein-tyrosine kinase that associates with the TCR zeta chain. Cell.

[28] Dubovsky JA, Beckwith KA, Natarajan G, Woyach JA, Jaglowski S, Zhong Y, Hessler JD, Liu T-M, Chang BY, Larkin KM, Stefanovski MR, Chappell DL, Frissora FW (2013). Ibrutinib is an irreversible molecular inhibitor of ITK driving a Th1-selective pressure in T lymphocytes. Blood.

[29] Da Roit F, Engelberts PJ, Taylor RP, Breij ECW, Gritti G, Rambaldi A, Introna M, Parren PWHI, Beurskens FJ, Golay J (2014). Ibrutinib interferes with the cell-mediated anti-tumour activities of therapeutic CD20 antibodies: implications for combination therapy. Haematologica.

[30] Braselmann S, Taylor V, Zhao H, Wang S, Sylvain C, Baluom M, Qu K, Herlaar E, Lau A, Young C, Wong BR, Lovell S, Sun T (2006). R406, an orally available spleen tyrosine kinase inhibitor blocks fc receptor signaling and reduces immune complex-mediated inflammation. J Pharmacol Exp Ther.

[31] Sharman J, Hawkins M, Kolibaba K, Boxer M, Klein L, Wu M, Hu J, Abella S, Yasenchak C (2015). An open-label phase 2 trial of entospletinib (GS-9973), a selective Syk inhibitor, in chronic lymphocytic leukemia. Blood.

[32] Huck J, Brake R, Tirrell S, He H, Theisen M, Yu J, Zhang M, Balani S, Atienza J, Vincent P, Manfredi M, Zalevsky J, Kannan K (2014). Antitumor activity of inhibiting SYK kinase with TAK-659, an investigational agent, in DLBCL models. J Clin Oncol.

[33] Petrich AM, Gordon LI, Infante JR, Nimeiri HS, Zhang B, Faucette S, Shou Y, Shih KC (2015). Ongoing, first-in-human, phase 1 dose-escalation study of investigational Syk inhibitor TAK-659 in patients with advanced solid tumours or lymphoma. Hematological Oncology.

[34] Rozovski U, Wu JY, Harris DM, Liu Z, Li P, Hazan-Halevy I, Ferrajoli A, Burger JA, O'Brien S, Jain N, Verstovsek S, Wierda WG, Keating MJ (2014). Stimulation of the B-cell receptor activates the JAK2/STAT3 signaling pathway in chronic lymphocytic leukemia cells. Blood.

[35] Suljagic M, Longo PG, Bennardo S, Perlas E, Leone G, Laurenti L, Efremov DG (2010). The Syk inhibitor fostamatinib disodium (R788) inhibits tumor growth in the Eμ- TCL1 transgenic mouse model of CLL by blocking antigen-dependent B-cell receptor signaling. Blood.

[36] Brown JR, Byrd JC, Coutre SE, Benson DM, Flinn IW, Wagner-Johnston ND, Spurgeon SE, Kahl BS, Bello C, Webb HK, Johnson DM, Peterman S, Li D (2014). Idelalisib, an inhibitor of phosphatidylinositol 3-kinase p110δ, for relapsed/refractory chronic lymphocytic leukemia. Blood.

[37] Ponader S, Chen S-S, Buggy JJ, Balakrishnan K, Gandhi V, Wierda WG, Keating MJ, O'Brien S, Chiorazzi N, Burger JA (2012). The Bruton tyrosine kinase inhibitor PCI-32765 thwarts chronic lymphocytic leukemia cell survival and tissue homing *in vitro* and *in vivo*. Blood.

[38] Herman SEM, Gordon AL, Wagner AJ, Heerema NA, Zhao W, Flynn JM, Jones J, Andritsos L, Puri KD, Lannutti BJ, Giese NA, Zhang X, Wei L (2010). Phosphatidylinositol 3-kinase-δ inhibitor CAL-101 shows promising preclinical activity in chronic lymphocytic leukemia by antagonizing intrinsic and extrinsic cellular survival signals. Blood.

[39] Herman SEM, Gordon AL, Hertlein E, Ramanunni A, Zhang X, Jaglowski S, Flynn J, Jones J, Blum KA, Buggy JJ, Hamdy A, Johnson AJ, Byrd JC (2011). Bruton tyrosine kinase represents a promising therapeutic target for treatment of chronic lymphocytic leukemia and is effectively targeted by PCI-32765. Blood.

[40] Hoellenriegel J, Meadows SA, Sivina M, Wierda WG, Kantarjian H, Keating MJ, Giese N, O'Brien S, Yu A, Miller LL, Lannutti BJ, Burger JA (2011). The phosphoinositide 3′-kinase delta inhibitor, CAL-101, inhibits B-cell receptor signaling and chemokine networks in chronic lymphocytic leukemia. Blood.

[41] Kohrt HE, Sagiv-Barfi I, Rafiq S, Herman SEM, Butchar JP, Cheney C, Zhang X, Buggy JJ, Muthusamy N, Levy R, Johnson AJ, Byrd JC (2014). Ibrutinib antagonizes rituximab-dependent NK cell-mediated cytotoxicity. Blood.

[42] Chou TC (2010). Drug combination studies and their synergy quantification using the Chou-Talalay method. Cancer Res.

